# Association of perioperative adverse events with subsequent therapy and overall survival in patients with WHO grade III and IV gliomas

**DOI:** 10.3389/fonc.2022.959072

**Published:** 2022-09-28

**Authors:** Lorenz Weber, Luis Padevit, Timothy Müller, Julia Velz, Flavio Vasella, Stefanos Voglis, Dorothee Gramatzki, Michael Weller, Luca Regli, Johannes Sarnthein, Marian Christoph Neidert

**Affiliations:** ^1^ Department of Neurosurgery, University Hospital and University of Zurich, Zurich, Switzerland; ^2^ Department of Neurology, University Hospital and University of Zurich, Zurich, Switzerland; ^3^ Department of Neurosurgery, Kantonsspital St. Gallen, St. Gallen, Switzerland

**Keywords:** adverse events, complications, glioblastoma, treatment delay, subsequent therapy, high grade glioma, maximum-safe-resection, neurosurgery

## Abstract

**Background:**

Maximum safe resection followed by chemoradiotherapy as current standard of care for WHO grade III and IV gliomas can be influenced by the occurrence of perioperative adverse events (AE). The aim of this study was to determine the association of AE with the timing and choice of subsequent treatments as well as with overall survival (OS).

**Methods:**

Prospectively collected data of 283 adult patients undergoing surgery for WHO grade III and IV gliomas at the University Hospital Zurich between January 2013 and June 2017 were analyzed. We assessed basic patient characteristics, KPS, extent of resection, and WHO grade, and we classified AE as well as modality, timing of subsequent treatment (delay, interruption, or non-initiation), and OS.

**Results:**

In 117 patients (41%), an AE was documented between surgery and the 3-month follow-up. There was a significant association of AE with an increased time to initiation of subsequent therapy (p = 0.005) and a higher rate of interruption (p < 0.001) or non-initiation (p < 0.001). AE grades correlated with time to initiation of subsequent therapy (p = 0.038). AEs were associated with shorter OS in univariate analysis (p < 0.001).

**Conclusion:**

AEs are associated with delayed and/or altered subsequent therapy and can therefore limit OS. These data emphasize the importance of safety within the maximum-safe-resection concept.

## Introduction

The current standard treatment for WHO grade III and IV gliomas is maximum safe resection followed by radiotherapy plus/minus concomitant and maintenance alkylating-based chemotherapy (1). Perioperative adverse events (AEs) are an important and sensitive parameter in measuring management quality of the perioperative period (2, 3). Importantly, AEs occur frequently during and after neurosurgical interventions and are associated with poor functional status (4–6).

AE are considered to affect the course of further therapy and might lead to non-initiation, interruption, or delay of subsequent therapy and eventually to worse overall survival (OS) (6, 7). However, the association of treatment delay of therapy and OS remains controversial. Although the relevance of the exact timing of subsequent therapy remains uncertain, a systemic review (8) and different studies (9–12) seem to agree that a moderate delay of subsequent chemoradiotherapy does not have a detrimental effect on OS, but a delay of more than 42 days after surgery might be associated with worse OS.

Here, we analyzed the association between perioperative AE and both the timing of subsequent therapy as well as OS.

## Methods

### Study design and patient selection

We prospectively collected data from the patient registry of the Department of Neurosurgery at the University Hospital Zurich (5). We screened all patients with initial surgery for anaplastic astrocytoma (WHO grade III) or glioblastoma (WHO grade IV) for eligibility—between January 2013 and June 2017. All adult patients with complete clinical data regarding the timing and modality of subsequent therapy and the occurrence of AE were included. AE were defined as any deviation from the normal and uneventful postoperative course. All data were collected by neurosurgeons at the time of hospital admission, surgery, hospital discharge, and at the time of each outpatient follow-up, which is commonly performed at 3 months postoperative for all patients undergoing cranial tumor surgery and continued on an individual basis afterward. All discharge reports are verified by the respective surgeon himself. Furthermore, all AE are validated at the monthly department meeting and at the monthly morbidity and mortality meeting to guarantee an accurate collection of data. Deaths before June 2019 were ascertained, and patients were censored at the last date they were stated to be alive.

### Treatment

All included patients underwent initial neurosurgical intervention for WHO grade III or IV glioma. The extent of resection (EOR) varied between biopsy-only, partial resection <98%, and gross total resection ≥98%. The protocol of subsequent therapy during the study period was based on the EANO guideline from 2014 (13). The standard radiotherapy dose was usually 60 Gray where the fractions varied according to clinical patient characteristics. First-line chemotherapy for glioblastoma during the study period was concomitant temozolomide during radiotherapy followed by 6 cycles of maintenance temozolomide. In case of progression or relapse under the first-line treatment with temozolomide, second-line chemotherapeutics (mostly CCNU) or bevacizumab were used.

### Study variables

Parameters extracted from the patient registry were age, sex, functional state, EOR, WHO grade, length of hospital stay (LOS), procedure and timing of subsequent therapy, any delay or interruption or non-initiation of the subsequent therapy protocol, the occurrence of AE, and OS. The patients’ functional state was quantified using the Karnofsky Performance Status (KPS). A worsened functional state was defined as a decrease in the KPS 3 months postoperatively compared to the preoperative KPS. EOR was assessed by three-dimensional volume measurements of pre- and <72 h postoperative MRI scans using Smartbrush application of Brainlab Elements, Brainlab AG Munich. In case of lacking MRI data, EOR was not further evaluated.

Perioperative AE with its corresponding clinical diagnosis were assessed using the Clavien–Dindo Grade (CDG) classification and graded accordingly (**Table 1**) (14).

**Table 1:** Frequency of clinical diagnoses as cause of an adverse event (AE).

Frequency of diagnoses of AE		
Diagnosis	Number of AE	CDG grade (number of AE)
Wound healing disorder/dehisence	3	IIIa (1), **IIIb** (2)
Surgical site infection	7	II (3), **IIIb** (4)
Secoundary bleeding	9	**I** (8), IVa (1)
Stroke	14	**I** (9), II (4) IVa (1)
New neurological deficit	55	**I** (48), II (6), IIIb (1)
First-time epilepsy	19	**II** (19)
Died within 30 days	5	**V** (5)
Thrombosis	2	**II** (2)
Pulmonary embolism	7	**II** (6), IIIa (1)
Urinary tract infections	16	**II** (16)
Pneumonia	13	**II** (13)
Hernation	1	**II** (1)
Others		
Delir	3	**II** (3)
Shunt dysfunction^*^	1	**IIIb** (1)
Meningitis	1	**IIIb** (1)
SIADH	1	**II** (1)
Myocardial infarction	1	**IIIa** (1)
Hypotension	1	**II** (1)
External otitis	1	**II** (1)
Corneal erosion	1	**I** (1)
Urinary tract disorder	1	**IIIa** (1)
Liver disorder	2	**II** (2)
**Total number of AE**	**164**	

A total of 164** **AE occurred in 117 patients, whereby in some cases more than one AE occurred. In the third column, the resulting grade of AE (Clavien–Dindo–Grade (CDG)) is displayed. The most frequent CDG per diagnoses are marked in bold. ^*^Patient suffering from an entrapped temporal horn due to tumor occlusion with implantation of a ventriculoperitoneal shunt during biopsy which needed revision surgery due to an non-functional gravitational device. The underlying diagnosis that lead to AE are shown.

We defined the variable “altered subsequent therapy” as the occurrence of either a delay, interruption, or non-initiation of the subsequent therapy. The time to initiation of subsequent therapy was defined as duration between the intervention and the beginning of subsequent therapy. A delay in subsequent therapy was defined as a duration of >42 days until initiation. Any interruptions of therapy were additionally registered and defined as any break in the protocol of subsequent therapy. The non-initiation of subsequent therapy was defined as the absence of subsequent chemo- and radiotherapy.

OS was defined as the time from surgery until death or the last date when the patient was known to be alive.

### Statistical methods

The patient registry is based on FileMaker Pro version 13.0. For statistical analysis, IBM SPSS Statistics v24 and MATLAB R2019a were used. A p-value of ≤0.05 was considered statistically significant. Continuous data were analyzed with two-tailed Mann–Whitney U tests and Student’s t-tests and categorical data with two-sided Pearson χ^2^ tests, correlations using Spearman correlation tests, OS using Kaplan–Meier and log-rank tests, and multivariate analysis using Cox’s proportional hazard model. Hazard ratios (HR) are given with 95% confidence intervals (CI).

### Ethics

The local ethics committee (Kantonale Ethikkommission) approved prospective data collection in the patient registry and waived patient consent due to the observational nature of the study (PB-2017-00093). The study was registered at clinicaltrials.gov (NCT01628406).

## Results

### Patient characteristics and outcome data

Two hundred eighty-three patients were included in our study. The main clinical characteristics and outcome data of the study group stratified for AE are depicted in **Table 2**. A statistically significant difference in patient characteristics between patients with AE and patients without AE was seen for median age, LOS, and therapy groups. While the median and the interquartile range of the preoperative KPS scores are identical, the two distributions differ significantly as well as at discharge and at 3 months postoperatively. For the outcome data, a statistically significant difference between the subgroups was found for worsened functional state, altered subsequent therapy, delay in subsequent therapy, interruption, non-initiation, and median OS.

**Table 2:** Patient characteristics and outcome variables stratified for AE.

Patient characteristics and outcome variables stratified for AE				
Variables	Overall (n = 283)	No AE (n = 166)	AE (n = 117)^a^	p-value
Median age, years (IQR)	63 (52-72)	61 (52-69)	67 (54-74.5)	0.005^c*^
Female, n (%)	95 (34)	58 (35)	37 (32)	0.561^d^
Glioblastoma, n (%)	245 (87)	139 (84)	106 (91)	0.095^d^
EOR, n (%)				
Biopsy only	89 (31)	47 (28)	42 (36)	0.176^d^
Partial resection (EOR <98%)	114 (40)	72 (43)	42 (36)	0.207^d^
Gross total resection (EOR ≥98%)	69 (24)	42 (25)	27 (23)	0.668^d^
Unclear extent of resection	11 (4)	5 (3)	6 (5)	0.364^d^
Therapy groups, n (%)				
CRT	174 (62)	116 (70)	58 (50)	<0.001^d*^
CT	33 (12)	22 (13)	11 (9)	0.320^d^
RT	45 (16)	22 (13)	23 (20)	0.147^d^
noT	31 (11)	6 (4)	25 (21)	<0.001^d*^
Median KPS score preoperatively, % (IQR)	80 (70-90)	80 (70-90)	80 (70-90)	0.009^c*^
Median KPS score at discharge, % (IQR)	80 (70-90)	90 (80-90)	70 (50-80)	<0.001^c^*
Median KPS score at 3 months postoperatively, % (IQR)	80 (60-90)	90 (80-90)	60 (15-80)	<0.001^c^*
Worsened functional state, n (%)	130 (46)	56 (33)	74 (63)	<0.001^d^*
Mean time to initiation of subsequent therapy, days (SD)^b^	32 (12)	30 (8.5)	35 (17)	0.005^e^*
Length of hospital stay, days (SD)	8 (5)	7 (3)	9 (6)	<0.001^c^*
Altered subsequent therapy, n (%)	82 (29)	23 (13)	59 (50)	<0.001^d^*
Delay (>42 days) in subsequent therapy^b^	28 (11)	12 (7.5)	16 (17)	0.016^d^*
Interruption	29 (10)	5 (3.0)	24 (21)	<0.001^d^*
Non-initiation	31 (11)	6 (3.6)	25 (21)	<0.001^d^*
Median overall survival, months (95% CI)	13 (11.3-14.7)	17 (14.5-19.5)	9 (6.7-11.3)	<0.001^f^*

AE, adverse event; EOR, extent of resection; CRT, chemoradiotherapy; CT, chemotherapy; RT, radiotherapy; noT, no other therapy than best supportive care; KPS, Karnofsky Performance Status; IQR, interquartile range; SD, standard deviation; CI, confidence interval. ^a^At least one AE from surgery to 3 months postoperatively. ^b^At least one AE prior to start of subsequent therapy. ^c^Mann–Whitney U test. ^d^χ^2^ test. ^e^Student’s t-test. ^f^Log-rank test.

*Statistically significant. All statistically significant p-values are highlighted in bold.

### AE were associated with delay and altered subsequent treatment

**Figure 1:**
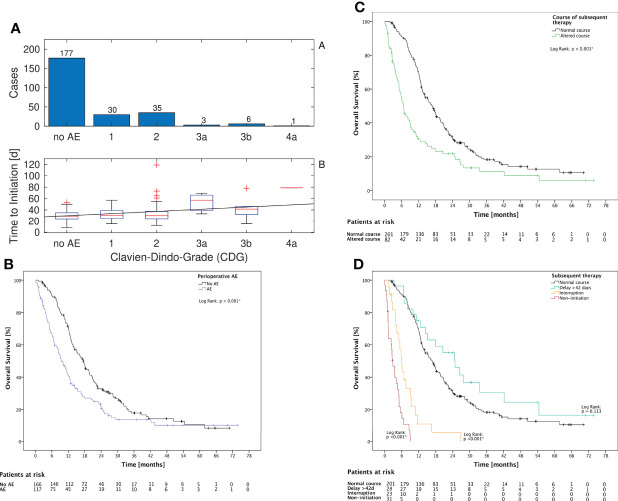
CDG grade, KPS, and subsequent therapy **(A)**: Of all 252 cases with any subsequent therapy, in 75 cases an AE prior to beginning of subsequent therapy was noted. The time to initiation and the CDG grade were correlated with Spearman’s rho = 0.13 (p = 0.038). The linear fit has a slope of 3.9 days per increment of CDG. **(B)**: The occurrence of AE until 3 months postoperatively is associated with a significantly lower OS in Log Rank test (p < 0.001) **(C)**: The occurrence of altered subsequent therapy is associated with a significantly lower OS in log-rank test (p < 0.001) **(D)**: The subgroups with interruption or non-initiation of subsequent therapy had both a significant decreased OS (p < 0.001). The subgroup with delay showed no significant association with OS (p = 0.113).

The mean time to initiation was significantly higher for patients with AE prior to therapy (p = 0.005, Mann–Whitney U test) and correlated significantly with the grade of AE (p = 0.038, Spearman’s rho = 0.13, **Figure 1A**). Altered subsequent therapy in general and each of the underlying variables (either delay or interruption or non-initiation of the subsequent therapy) was more frequent in the subgroup of patients with AE (**Table 2**).

**Figure 2:**
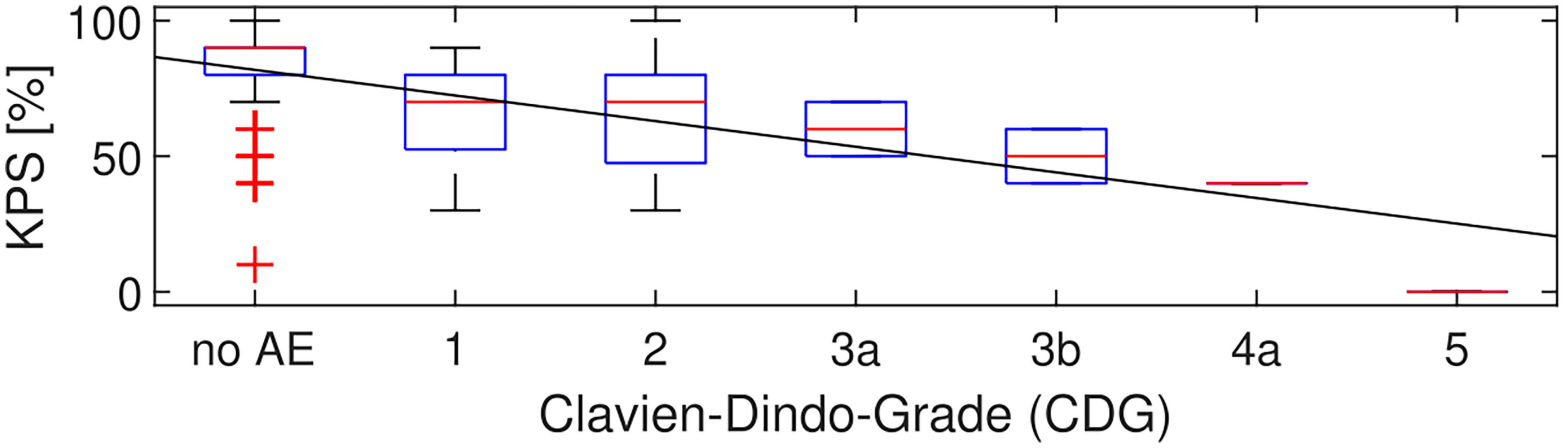
CDG grade and KPS. Over all 283 patients, in 78 patients at least one AE occurred before discharge. KPS and CDG at discharge were correlated with Spearman’s rho = -0.41 (p < 0.001). The linear fit had a slope of -9.5 KPS points per increment of CDG.

Additionally, there was a significant correlation between the severity of CDG and KPS at discharge (p < 0.001, Spearman’s rho = -0.41) with a slope of -9.5 KPS points per increment of CDG in the linear fit (**Figure 2**).

### AE were associated with worse OS

OS for patients with AE was shorter (p < 0.001, Mann–Whitney U test) (**Figure 1B**). However, there was no significant correlation between the CDG of the AE and OS (p = 0.063, Mann–Whitney U test). Altered subsequent therapy and its underlying variables “interruption” and “non-initiation” were also associated with a significant decrease in OS (**Figures 1C, D**). However, the delay itself was not associated with a change in OS (p = 0.113, **Figure 1D**). LOS was negatively correlated with OS (Spearman’s rho = -0.152, p = 0.011).

A multivariate Cox proportional hazard model was conducted to estimate the association of occurrence of AE and altered subsequent therapy with OS (adjusted for confounders age, sex, WHO grade, preoperative KPS, and EOR). There was a trend toward shorter OS in patients with AE with HR = 1.32 (CI 0.96–1.77), although this did not reach statistical significance (p = 0.063). Altered subsequent therapy had a stronger and significant association with shorter OS (HR = 1.97, CI 1.44–2.69, p < 0.001) (**Table 3**).

**Table 3:** Predictor of overall survival (OS).

Prognostic factors of overall survival				
Variables	Coefficient Exp(B)	SE	95% CI	p-value
**Occurrence of AE from surgery to 3 months postoperatively**	**1.32**	**0.15**	**0.99 - 1.77**	**0.063**
**Altered subsequent therapy**	**1.97**	**0.16**	**1.44 - 2.69**	< 0.001*
Age	1.04	0.01	1.03-1.05	< 0.001*
Male sex	0.99	0.14	0.75-1.31	0.949
Tumor grade (WHO grade)	2.28	0.27	1.33-3.89	0.003*
Preoperative KPS	0.99	<0.01	0.98-0.99	0.003*
Extent of resection (reference category *biopsy only*)				
Partial resection	0.65	0.16	0.47-0.90	0.008*
Gross total resection	0.50	0.19	0.35-0.72	< 0.001*
Unclear extent of resection	0.79	0.36	0.39-1.59	0.514

The putative predicting factors for OS as AE and altered subsequent therapy were analyzed using a Cox proportional hazard model correcting for confounders. AE, adverse event; KPS, Karnofsky Performance Status; SE, standard error.

*Statistically significant. Prognostic factors of main interest are highlighted in bold.

## Discussion

There is only scarce information about the frequency and the exact clinical repercussions of AE in glioma surgery. The risk of AE in the resection of WHO grade III and IV glioma is relatively high due to the difficult balancing between a maximal tumor resection and new postoperative neurological deficits and other AE (6). Therefore, knowing the consequence of a perioperative AE is of utmost importance.

The impact of timing of subsequent therapy on OS is still unclear. Nevertheless, different studies indicate that a delay of >42 days should be avoided (8–12). We observed a significantly higher mean time to initiation of subsequent therapy and a corresponding higher rate of delay ≥42 days for patients with an AE. We also observed that AE with higher CDG scores were associated with a longer time to initiation. These findings emphasize that surgical AE can entrail a suboptimal timing and course of subsequent therapy. In addition, our findings indicate that patients with AE are less likely to receive the standard of care therapy; the rate of delay, interruption, and non-initiation is significantly higher.

Regarding OS, patients with AE had significantly decreased median survival in a univariate analysis in line with findings of previous studies (6, 7). In a multivariate analysis, patients with AE had a strongly increased hazard ratio adjusted for possible confounders, although the result was not significant, likely due to the relatively small size of the study group. The potential detrimental effect of AE on OS can be explained by the direct negative effect of AE on the patient’s health status, and furthermore by a higher rate of altered subsequent therapy. The association of non-initiation and interruption of subsequent therapy with decreased OS was clearly shown in this study. However, we did not find any evidence showing that a delay >42 days of subsequent therapy alone is associated with inferior OS.

As a retrospective analysis of prospectively collected data, our study is subjected to the general limitations of retrospective studies. Still, our outcome data was collected prospectively and modified retrospectively only in case of incompleteness, which minimized the risk of information bias. It has to be taken into account that some patients already started subsequent therapy within the 3-month period in which AE were measured. Thus, it cannot be excluded that some of the AE might also be due to subsequent therapy and are not related to surgery. Although the majority of AE occurred in close temporal proximity to the surgical intervention and measured AE are almost exclusively logically attributed to surgery rather than subsequent therapy, this limitation has to be considered. Furthermore, one must keep in mind that the lower patient age and better KPS preoperatively for patients without an AE as well as molecular tumor markers play a major role in prognosis and OS in high-grade glioma patients and can therefore interfere with our results, which poses another source of limitation for our study.

Future projects should incorporate the new 2021 WHO classification of brain tumors and should also reflect measurements for AE severity such as the Therapy–Disability–Neurology Grading as well as measures for the presurgical factors such as initial patient status or the complexity of the tumor surgery such as the Milan Complexity Score (15).

In conclusion, our results support the hypothesis that AE reduce the rate of successful and uninterrupted chemoradiotherapy and ultimately limit OS. While this association seems intuitively true, our study now supports it with data from a large patient cohort. In the sensitive balancing act between maximal resection and postoperative neurological function, our study quantifies the risk of causing AE and by that a possible impairment of the course of subsequent therapy and OS. Therefore, these findings have implications in risk stratification and quality management of WHO grade III and IV glioma surgery.

## Data availability statement

The raw data supporting the conclusions of this article will be made available by the authors, without undue reservation.

## Ethics statement

This study was reviewed and approved by Kantonale Ethikkommission Zürich, Stampfenbachstrasse 121, 8090 Zürich, Switzerland. The ethics committee waived the requirement of written informed consent for participation due to the observational nature of the study.

## Author contributions

Study design: LW, JS, MN. Data acquisition: LW, TM, JV, FV, SV, LP, JS, MN. Statistical analysis: LW, LP. Interpretation: all authors. Writing of original draft: LW, L P. Review and revision: all authors. All authors contributed to the article and approved the submitted version.

## Conflict of interest

The authors declare that the research was conducted in the absence of any commercial or financial relationships that could be construed as a potential conflict of interest.

## Publisher’s note

All claims expressed in this article are solely those of the authors and do not necessarily represent those of their affiliated organizations, or those of the publisher, the editors and the reviewers. Any product that may be evaluated in this article, or claim that may be made by its manufacturer, is not guaranteed or endorsed by the publisher.
